# Evaluation of the Antioxidant, Cytotoxicity, Antibacterial, Anti-Motility, and Anti-Biofilm Effects of *Myrothamnus flabellifolius* Welw. Leaves and Stem Defatted Subfractions

**DOI:** 10.3390/plants13060847

**Published:** 2024-03-15

**Authors:** Mashilo Mash Matotoka, Peter Masoko

**Affiliations:** Faculty of Science and Agriculture, Department of Biochemistry, Microbiology and Biotechnology, University of Limpopo, Private Bag X1106, Sovenga 0727, South Africa; mashilo.matotoka@ul.ac.za

**Keywords:** medicinal plants, antioxidant, antibacterial, anti-motility, anti-biofilm

## Abstract

The formation of biofilms underscores the challenge of treating bacterial infections. The study aimed to assess the antioxidant, cytotoxicity, antibacterial, anti-motility, and anti-biofilm effects of defatted fractions from *Myrothamnus flabellifolius* (resurrection plant). Antioxidant activity was assessed using DPPH radical scavenging and hydrogen peroxide assays. Cytotoxicity was screened using a brine shrimp lethality assay. Antibacterial activity was determined using the micro-dilution and growth curve assays. Antibiofilm potential was screened using the crystal violet and tetrazolium reduction assay. Liquid–liquid extraction of crude extracts concentrated polyphenols in the ethyl acetate and n-butanol fractions. Subsequently, these fractions had notable antioxidant activity and demonstrated broad-spectrum antibacterial activity against selected Gram-negative and Gram-positive bacteria and *Mycobacterium smegmatis* (MIC values < 630 μg/mL). Growth curves showed that the bacteriostatic inhibition by the ethyl acetate fractions was through the extension of the lag phase and/or suppression of the growth rate. The sub-inhibitory concentrations of the ethyl acetate fractions inhibited the swarming motility of *Pseudomonas aeruginosa* and *Klebsiella pneumoniae* by 100% and eradicated more than 50% of *P. aeruginosa* biofilm biomass. The polyphenolic content of *M. flabellifolius* plays an important role in its antibacterial, anti-motility, and antibiofilm activity, thus offering an additional strategy to treat biofilm-associated infections.

## 1. Introduction

Bacteria cause numerous human infections. Gram-negative bacteria (GNB) are notorious human pathogens that can invade the bloodstream, digestive tract, nervous, and urinary systems [1]. On the other hand, Gram-positive bacteria (GPB) exert pressure on public health due to their significant antibiotic resistance [2]. Recently, there has been a reported increase in atypical mycobacterial infections [3]. These non-tuberculosis pathogens are associated with skin, soft tissue, lymph nodes, and pulmonary infection [3].

The upsurge of antibiotic resistance by human pathogens has proliferated mortality and morbidity rates across the globe [4]. This underlines the need for alternative antibacterial drugs that can exert different mechanisms of action. Bacteria use virulence factors to initiate infection and evade the host’s immune defences [5]. Notable virulence factors include biofilm, quorum sensing, motility, toxins, adherence factors, and polysaccharide capsules [6,7]. An alternative approach to curb the increase in bacterial infections and antibiotic resistance is anti-virulence therapy, which involves the interference of the upregulation and functionality of virulence factors without inhibiting bacterial growth. The advantage of this approach is that it causes less selective pressure for the development of resistance genes and may potentially reduce the rate of emerging antibiotic-resistant strains [7,8]. Moreover, anti-virulence therapy may reduce the severity of infection by enabling the host to have an effective immunity to the invading bacteria [9].

A biofilm is a group of microorganisms that adhere to biotic or abiotic surfaces and form microcolonies that are enclosed by an extracellular polymeric substance (EPS). The EPS comprises macromolecules such as proteins, carbohydrates, lipids, and extracellular deoxyribonucleic acid [10], which confer protection against ultraviolet radiation and alterations in pH [11]. Mature biofilms are challenging to eliminate; thus, intervening in the early stage of biofilm formation can be a solution [12]. A majority of chronic or persistent bacterial infections (80%) were attributed to the formation of biofilms [13]. Conventional antibiotics are active against planktonic cells but do not effectively sterilize the central region of biofilms and this leads to recurring infections [14].

*Myrothamnus flabellifolius* Welw. (Myrothamnaceae family) is a desiccant-tolerant, woody, and shrubby plant that has green fan-like shaped leaves that have a waxy abaxial surface and usually fold along the stem [15]. It is common for *M. flabellifolius* to grow on shallow ground; however, it can also survive on rocky slopes due to its extensive root system extending into crevices. This allows the plant to survive dehydration for long periods of time. The colour of the leaves during desiccation changes from green to brown [16,17]. The plant is native to southern Africa and is extensively spread out in Botswana and numerous provinces in South Africa, which include the North West, Mpumalanga, Gauteng, KwaZulu-Natal, and Limpopo [18,19]. This plant is commonly known as *Umfavuke* (isiNdebele), *Uvukabafile* (isiZulu), *Moritela Tshwene* (Setswana), and *Mufandichimuka* (Shona) [20]. Bapedi healers use it to treat sexually transmitted infections [21] and chest pains [22]. In southern Zimbabwe and Botswana, locals boil ground leaves and twigs to prepare tea that is used to treat respiratory infections [23,24]. The aerial parts of the plant have also been used for the treatment of asthma, inflammation, epilepsy, the heart, wounds, backaches, diabetes, kidney ailments, hypertension, haemorrhoids, gingivitis, shingles, stroke, and skin conditions [21,25,26]. *M. flabellifolius* leaves have flavonoids, alkaloids, terpenoids, proanthocyanidines, condensed tannins, flavan-3-ols, saponins, and phytosterols [27,28]. The focus of this work was to investigate the biological activities of defatted polyphenolic-enriched sub-factions of *M. flabellifolius* leaves and stems. More notably, the anti-motility and anti-biofilm activities have previously not been investigated. 

## 2. Results and Discussion

### 2.1. Extraction Yield 

**Figure 1:**
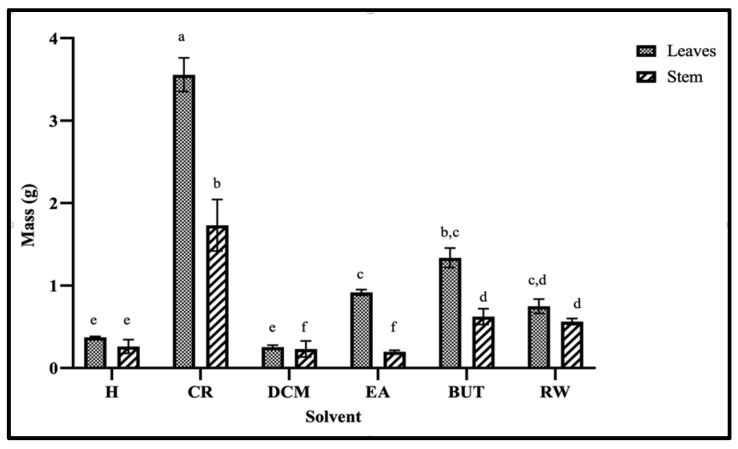
Extraction yield from 5 g of *Myrothamnus flabellifolius* leaves and stems samples. H: hexane. CR: 70% acidified acetone crude extract. DCM: dichloromethane. EA: ethyl acetate. BUT: butanol. RW: residual water. Data in the bar chart is expressed as mean ± standard deviation. Values with different letter superscripts in a column are significantly different (*p* < 0.05), and same letter represents non-significance (*p* > 0.05).

Organic solvent and aqueous extracts of *M. flabellifolius* leaves have been reported to possess various bioactive phenolic compounds [18,29]. To increase the yield of the extraction of polyphenols, the plant material was extracted with 70% acidified acetone. Hexane was used for defatting the crude extract by removing extremely non-polar and interfering components such as chlorophyll, long chained fatty acids, and wax constituents. The defatted 70% acidified acetone extracts were regarded as crude extracts. The highest crude extract yield was obtained from the leaves (3.56 g). The stem crude extract was significantly lower (1.73 g) than the leaves crude extract. Of the subfractions obtained from the leaf crude extract, the n-butanol had the highest obtained mass (1.34 g), followed by the ethyl acetate fraction (0.92 g) (Figure 1). The stem fractions generally had lower extracted mass per the corresponding solvent. Extraction efficiency plays a crucial role in ensuring the quality and economic viability of plant extract-based products across various industries; therefore, the leaves can serve as a more sustainable source of bioactive phytochemicals in *M. flabellifolius* than the stem parts.

### 2.2. Phytochemical Analysis

**Figure 2:**
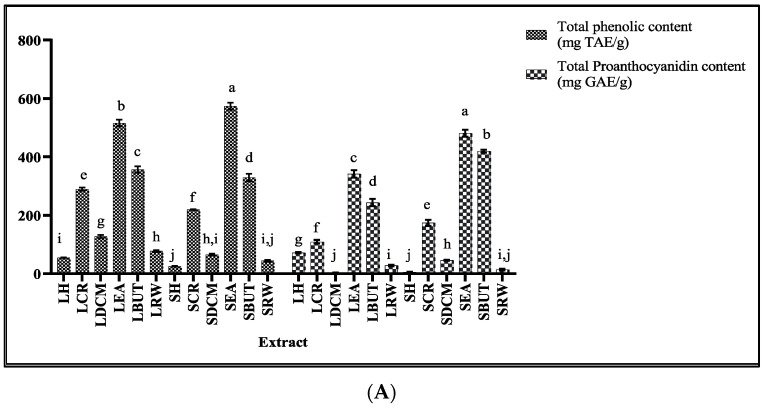

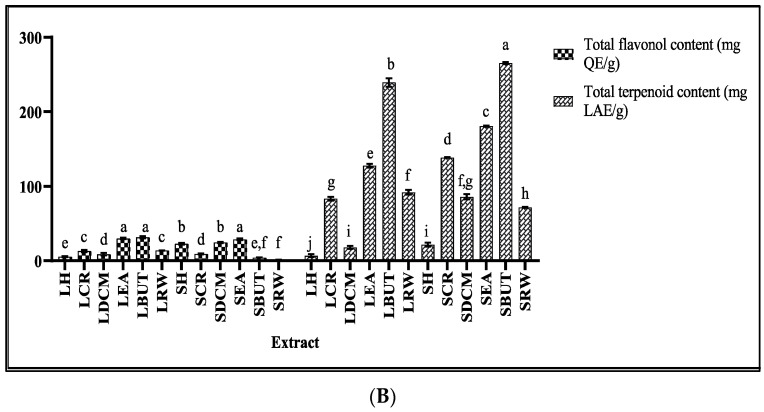
Quantification of total phenolic and proanthocyanidin contents (**A**) and total flavonol and terpenoid contents (**B**) of *Myrothanmus flabellifolius* leaves and stem extracts. LH: leaf hexane. LCR: leaf crude. LDCM: leaf dichloromethane. LEA: leaf ethyl acetate. LBUT: leaf butanol. LRW: leaf residual water. SH: stem hexane. SCR: stem crude. SDCM: stem dichloromethane. SEA: stem ethyl acetate. SBUT: stem butanol. SRW: stem residual water. Data in the bar chart is expressed as mean ± standard deviation. Values with different letter superscripts in a column are significantly different (*p* < 0.05), and same letter represents non-significance (*p* > 0.05).

The quantification of the polyphenolic compounds showed that the ethyl acetate fractions (LEA and SEA) from both the leaves and stems had significantly higher total phenolic and proanthocyanidin contents (Figure 2). These results were in accordance with those reported by Anke et al. [30], where the ethyl acetate subfraction obtained from a defatted acetone–water extract yielded fractions enriched with proanthocyanidins, oligomeric proanthocyanidins, and flavan-3-ols [30]. In addition, 2,3-di-O-galloylarbutin was isolated from an ethyl acetate soluble fraction of an acetone/water crude extract of *M. flabellifolia* [31]. The total flavonol contents were detected in low amounts in the different fractions. The variability of the polyphenolics in *M. flabellefolius* was attributed to habitat differences [32]. Total terpenoid content was higher in the butanol fractions (LBUT and SBUT), followed by the ethyl acetate subfractions (LEA and SEA). Monoterpenes predominantly found in *M. flabellifolius* are pinocarvone and trans-pinocarveol [20]. The distribution of the different phytochemical groups demonstrated that the liquid–liquid extraction aggregated most of the bioactive compounds in the ethyl acetate and n-butanol subfractions. Polyphenols were associated with antioxidant, anti-inflammatory, antiproliferative, antimicrobial, and anti-mutagenic bioactivities [33].

### 2.3. Antioxidant Activity

Antioxidants are known to neutralize free radicals and have been linked to antibacterial and anti-biofilm formation activities [34]. The SEA and LEA subfractions had the lowest values EC_50_ values of 0.46–2.99 μg/mL and 0.13–7.57 μg/mL, respectively. This was significantly higher antioxidant activity than L-ascorbic acid (20.41 μg/mL) (Table 1). Aqueous–organic solvent extracts were previously reported to have notably high DPPH scavenging and hydrogen peroxide-reducing activities [35]. In addition, Bhebhe et al. [36] showed that aqueous extracts (herbal teas) of *M. flabellefolius* had significant antioxidant properties. The LEA (163.19 ± 0.90 μg/mL) and SEA (197.97 ± 0.32 μg/mL) had better hydrogen peroxide-reducing activity compared to L-ascorbic acid (364.2 ± 0.80 μg/mL). Hydrogen peroxide forms part of reactive oxygen species (ROS) that impair many cellular and mitochondrial biomolecules. The reduction of hydrogen peroxide limits the negative impacts of oxidative stress [37]. The high antioxidant activity appeared to be directly proportional to the high polyphenolic contents. 

### 2.4. Cytotoxicity

The assessment of toxicity of the fractions was derived from the previous toxicity profile of brine shrimps reported by Bussmann et al. [38]: where LC_50_ < 249 μg/mL was considered highly toxic, LC_50_ values between 250 and 499 μg/mL were considered to have moderate toxicity, LC_50_ values between 500 and 999 μg/mL had low toxicity, and LC_50_ values higher than 1000 μg/mL were non-toxic. All the extracts had LC_50_ > 500 μg/mL, and LBUT had the lowest LC_50_ value of (529.79 ± 1.09 μg/mL) (Table 2). Therefore, all the fractions had low cytotoxicity. The most non-cytotoxic fractions were the stem residual water extract (SRW) (2157.05 ± 0.77 μg/mL), followed by its crude extract (1430 ± 1.16 μg/mL). Polyphenol-enriched extracts of *M. flabellifolius* were reported to be non-cytotoxic (LC_50_, 50 μg/mL) against Vero cells (African green monkey kidney cells) [39]. In addition, Chukwuma et al. [18] demonstrated the protective effect of *M. flabellifolius* polyphenols against oxidative hepatic cell injury using Chang liver cells. These results corroborated the non-cytotoxicity of *M. flabellifolius* polyphenols. The literature on in vivo toxicology evaluation tests [40] is scarce.

### 2.5. Antibacterial Activity on Planktonic Cells

#### 2.5.1. Minimum Inhibitory Concentrations of Extracts

The essential oils of *M. flabellifolius* were reported to have camphor and eucalyptol that add flavour to commonly brewed caffeine-free teas made from the leaves [40]. Correspondingly, previous studies gave credence to the in vitro antioxidant activity [35,41] and the antimicrobial potential of *M. flabellufolius* essential oils [42,43]. Reports on the antibacterial and antibiofilm activities of the defatted polyphenolic fractions are not as robust. Minimum inhibitory concentration (MIC) is the most common approach to express antibacterial activity and represents the lowest concentration of a drug that inhibits microbial growth [44]. 

All the fractions from the leaves, except for the residual water subfraction (LRW), demonstrated notable antibacterial activity, where MIC values ≤ 630 μg/mL were determined against three GNB (Table 2). For the stem fractions, only the SDCM and residual water (SRW) had weak antibacterial activity against GNB. On the other hand, only LCR, LEA, SEA, and SBUT fractions had significant antibacterial activity against *Staphylococcus aureus* (MIC ≤ 630 μg/mL). Gram-negative bacteria are generally known to be more drug resistant than GPB due to the presence of an outer membrane [45]. Interestingly, our results generally showed that some fractions (LDCM, LBUT, and SCR) inhibited the test GPB (*S. aureus*) at higher concentrations than GNB. Gram-positive bacterial cell walls are composed of a thick peptidoglycan layer and lipoteichoic acids [46]. The MIC values of ethyl acetate and butanol fractions (LEA, SEA, LBUT, and SBUT) were 630 μg/mL against *M. smegmatis*. Given that traditional cultures around southern Africa use the plant to treat respiratory infections, the antimycobacterial activity of the fractions requires further evaluations against *Mycobacterium tuberculosis.* These results showed that the polyphenolic-enriched fractions of the stems and leaves of *M. flabellifolius* had broad-spectrum antibacterial activity. Relatively low selectivity indices (SI values) suggest that a test sample is toxic and cannot be used as a (herbal) drug, and SI values between 1 and 10 are generally moderate to non-cytotoxic. The subfractions generally demonstrated better selectivity of bacterial growth inhibition than brine-shrimp nauplii cytotoxicity. The highest selectivity values were obtained with SEA (SI: 5.9) followed by SCR (SI, 4.6) against *P. aeruginosa*. The lowest SI value (0.3) was obtained from LRW against *S. aureus* and *M. smegmatis*. The subfractions from both the leaves and stem demonstrated significant antibacterial activity with high selectivity to the bacterial cells.

#### 2.5.2. Growth-Kinetic Curves

**Figure 3:**
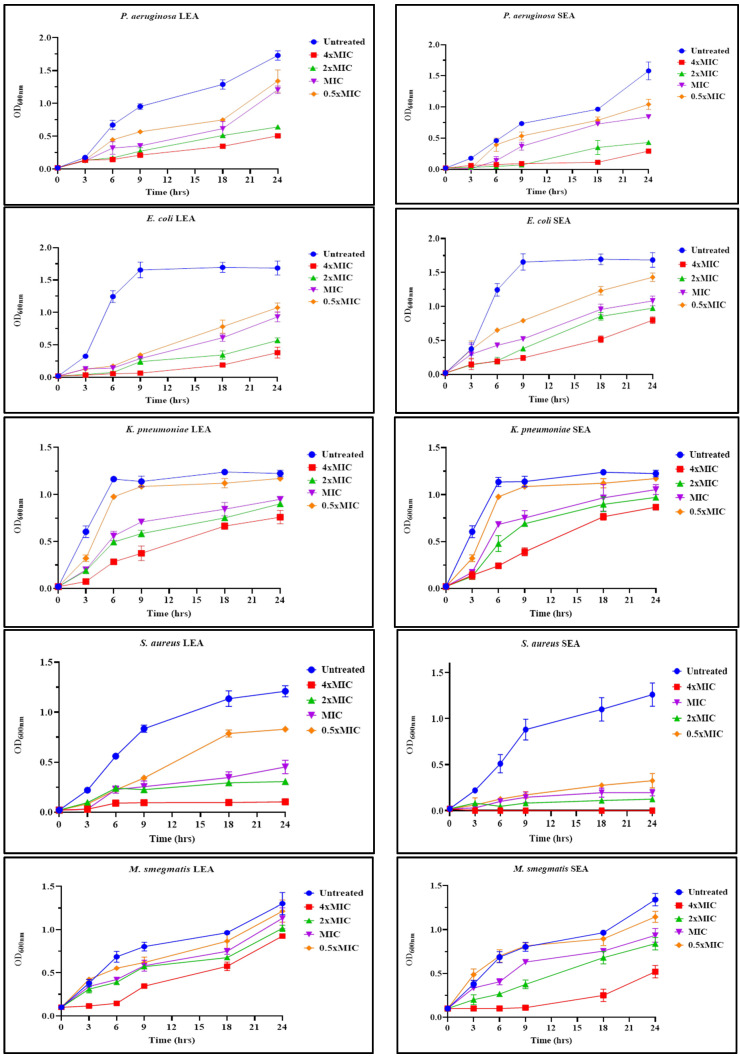
Growth curves of culture treated with different concentrations of ethyl acetate fractions of the leaves and stem. MIC: minimum inhibitory concentration, LEA: leaf ethyl acetate fraction, SEA: stem ethyl acetate fraction.

The growth-kinetic curve assay was conducted to investigate the kinetics involved in the antibacterial activity of LEA and SEA. The selection of LEA and SEA was based on their significant broad-spectrum antibacterial activity towards planktonic cells. The assessment of growth bacterial cells over 24 h was compared to an untreated control culture. Both LEA and SEA had concentration-dependent antibacterial activity. The treated cultures of all the test bacteria exhibited phenotypic growth that was generally different from the control culture in two main ways: (1) the lag phase of bacterial growth was lengthened and/or (2) the growth rate of the planktonic culture was decreased resulting in a lower final absorbance value (Figure 3). Similar bacterial growth-kinetic curve responses were previously reported by Jung et al. [47], in which *Caesalpinia sappan* L. extracts lengthened the lag phase of methicillin-resistant *S. aureus* by 2 h before the culture reached the exponential phase. Moreover, a crude extract of *Adiantum philippense* was reported to have extended the lag phase of *E. coli*, *S. aureus*, *P. aeruginosa*, and *S. flexneri* [48].

As the treatment time progressed, bacterial growth gradually recovered. This demonstrated that the antibacterial activity of the fractions was bacteriostatic. One possible explanation may be that the extracts exert their antibacterial activity by first entering the cytoplasm to be metabolised before growth impairment occurs. Therefore, when the concentrations of the active antibacterial components in the media decrease, the cells recover and continue with normal growth. It was further suggested that some of the antibacterial mechanisms of action of polyphenols involve a change in bacterial metabolism through the inhibition of enzymes such as oxidoreductases, lyases, and transfer enzymes [49].

### 2.6. Anti-Motility Screening

**Figure 4:**
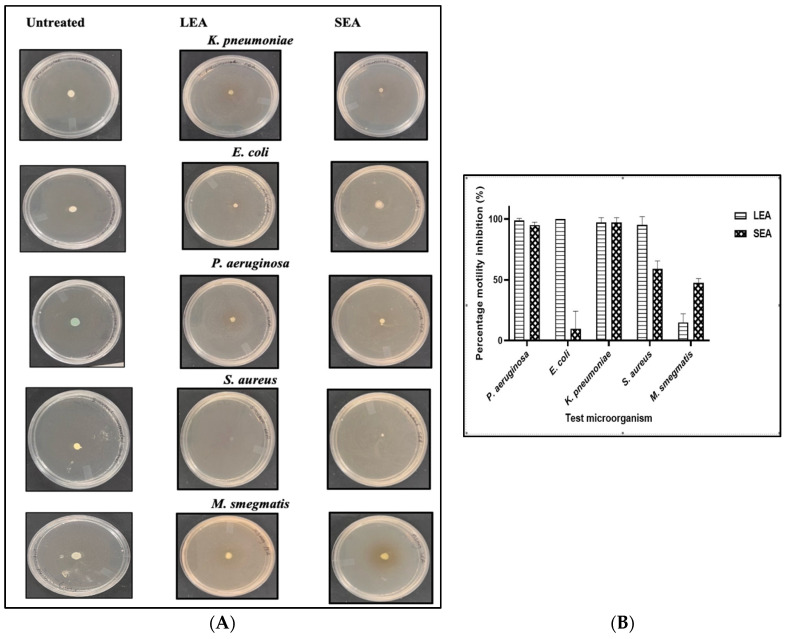
Anti-swarming effects of the leaves (LEA) and stem (SEA) ethyl acetate fractions against *K. pneumoniae*, *E. coli*, *P. aeruginosa*, *S. aureus*, and *M. smegmatis* (**A**) and their percentage motility inhibition (**B**). Values in (**B**) are expressed as mean ± standard deviation (SD) of duplicates.

The ethyl acetate fractions were further assessed for their potential to inhibit the motility of the test pathogens. Swarming contributes to the onset of early biofilm formation; thus, the inhibition of this motility may reduce the biofilm formation potential of bacteria [50]. The reduction of the size of the diameter at the point of inoculation by the fractions was measured and compared to an untreated control culture (Figure 4A). The LEA fractions inhibited 100% of *P. aeruginosa*, *E. coli*, *K. pneumoniae*, and *S. aureus* (Figure 4B). Other authors also reported that proanthocyanidin-rich extracts (100 μg/mL) of cranberry and pomegranate blocked the motility of *P. aeruginosa* [51]. The SEA generally had anti-motility activity. *M. smegmatis* motility was the most resistant to both fractions. *M. smegmatis* can spread on a surface by a sliding mechanism with the presence of glycopeptidolipids located in the outermost layer of the cell wall [52]. There is a scarcity of research assessing the anti-virulence potential of *M. flabellifolius*. The anti-motility potential of the LEA and SEA may indicate their interference with the early stages of biofilm formation.

### 2.7. Anti-Biofilm Screening

#### 2.7.1. Inhibition of Mature Biofilm

**Figure 5:**
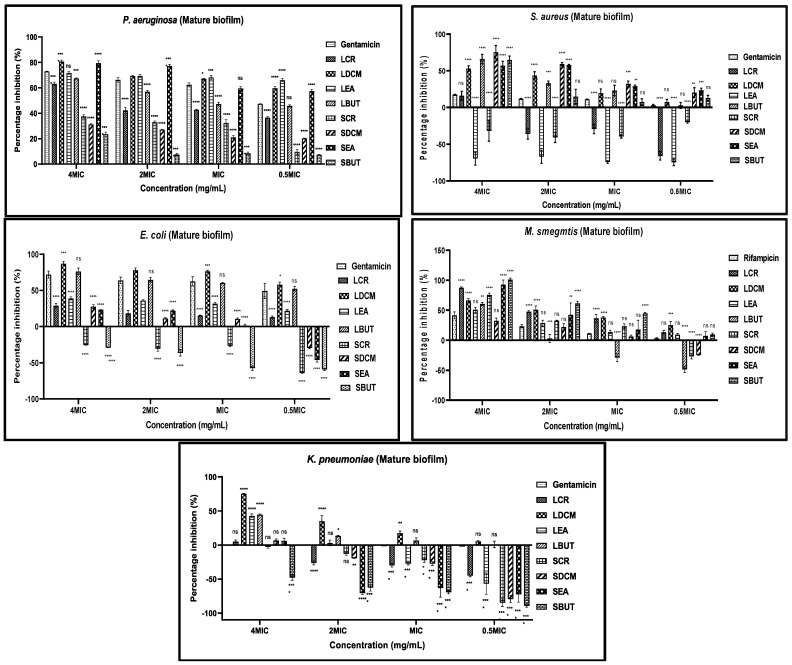
Antibiofilm activities of *M. flabellifolius* leaf and stem fractions by eradication of mature biofilms. LCR: leaf crude. LDCM: leaf dichloromethane. LEA: leaf ethyl acetate. LBUT: leaf butanol. SCR: stem crude. SDCM: stem dichloromethane. SEA: stem ethyl acetate. SBUT: stem butanol. Values expressed as mean ± standard deviation (SD) of triplicates; ns: not significant. (*): *p* < 0.05. (**): *p* < 0.01. (***): *p* < 0.001. (****): *p* < 0.0001.

Detecting and diagnosing biofilm-related infections can be more challenging due to their resilient nature. Mature biofilms exacerbate the development of antimicrobial resistance. Consequently, the efficacy of previously active antibiotics against planktonic cells becomes weakened. In line with other researchers, the eradication of more than 50% of the pre-formed biofilms was taken as notable anti-biofilm activity [53]. The mature biofilm of *P. aeruginosa* was the most susceptible among the selected GNB. It was further observed that the sub-minimum inhibitory concentrations (sub-MICs) (~1/2*MIC) of LDCM, LEA, and SEA fractions eradicated more than 50% of *P. aeruginosa* biofilm (Figure 5). Similarly, LDCM and LBUT eradicated more than 50% of *E. coli* biofilm at sub-MICs. This is significant because, at concentrations superior to or near the MIC, antimicrobials behave like toxins on susceptible bacterial cells. However, sub-MICs can induce diverse biological responses in bacteria associated with tolerance and biofilm formation [54]. The *K. pneumoniae* biofilms were the most resistant to eradication using both Gentamicin and fractions; only the LDCM at 4MIC was able to have substantial antibiofilm activity. The stem fractions generally showed weak activity against GNB biofilms but had significant activity against both *S. aureus* and *M. smegmatis* at higher concentrations (4MIC and 2MIC). The use of stem fractions to inhibit mature biofilms carries the risk of toxic side effects as higher concentrations increase the chances of toxicity and adverse effects when ingested [55]. The use of these stem fractions may be relevant to topical application against *M. smegmatis* and *S. aureus* infections involving the skin and wounds. Indeed, the potential use of polyphenols of *M. flabellifolius* for cosmetic and skin care was reported [39,43].

#### 2.7.2. Metabolic Activity of Mature Biofilms

**Figure 6:**
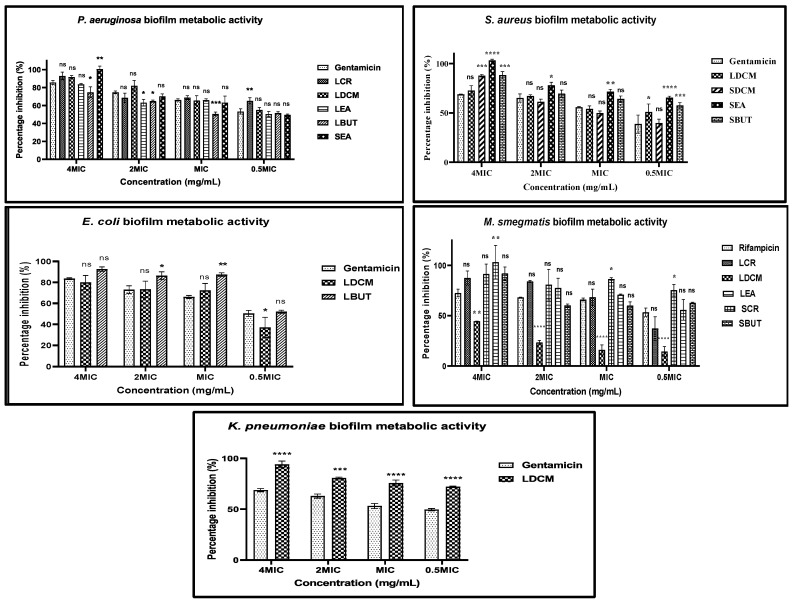
The effect of antibiofilm fractions on the metabolic activity of the biofilm biomass. LCR: leaf crude. LDCM: leaf dichloromethane. LEA: leaf ethyl acetate. LBUT: leaf butanol. SCR: stem crude. SDCM: stem dichloromethane. SEA: stem ethyl acetate. SBUT: stem butanol. Values expressed as mean ± standard deviation (SD) of triplicates. ns: not significant. (*): *p* < 0.05. (**): *p* < 0.01. (***): *p* < 0.001. (****): *p* < 0.0001.

One of the main drawbacks of the crystal violet assay is that the used dye has non-specific staining of the biofilm biomass. Therefore, there is no differentiation between viable cells, dead cells, and their associated EPS layer. An alternative approach to determine antibiofilm activity is to evaluate the metabolic activity commonly using tetrazolium salts. The MTT and resazurin were previously used to evaluate the extent of microbial growth in biofilms, where metabolically active cells reduce tetrazolium salt to a quantifiable purple formazan [56,57]. In this study, antibiofilm active extracts were assessed for their effect on the metabolic activity of the biofilms using INT. Like the eradication of mature biofilms, all the fractions had a concentration-dependent inhibition of the biofilm metabolic activities (Figure 6). In a different study, it was shown that gallic acid (phenolic acid) reduced the metabolic activity tested biofilms of *P. aeruginosa*, *S. aureus*, *L. monocytogenes*, and *E. coli* [57]. The inhibition of the metabolic activity by the polyphenolic-enriched fractions suggested that they were able to interact with the bacteria within the biofilms. Accordingly, it was reported that phenolic compounds can inhibit biofilms by interfering with regulatory pathways without significantly suppressing bacterial growth [58]. However, our results indicated that the mature biofilms of the test bacteria were eradicated by *M. flabellifolius* polyphenols by interfering with the biomass structure and inactivation of metabolic activity, making this plant a good source of antibiofilm compounds.

## 3. Materials and Methods

### 3.1. Plant Collection, Drying and Storage

The plant was collected from the Makgeng area (23.9416° S, 29.8254° E) in the Limpopo Province, South Africa, in March 2023 and identified by Dr. E. Bronwyn of the Larry Leach herbarium at the University of Limpopo (Voucher, SS 111). The leaves were separated from the stems and separately dried at ambient temperatures in an open laboratory environment with low humidity levels. The material was ground, and the fine powders (1.18 mm sieve used) were kept inside airtight glass jars.

### 3.2. Extraction

**Figure 7:**
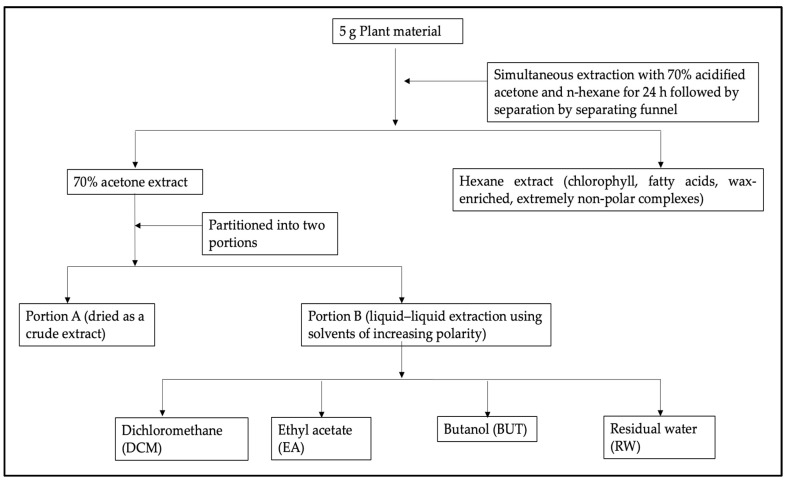
Flow chart showing the extraction and liquid–liquid fractionation of powdered leaves and stems of *Myrothamnus flabellifolius*.

The leaves and stem powdered material (5 g) was extracted using 70% acidified acetone and hexane simultaneously. To make up the 70% acidified acetone, 70 mL of acetone (Merk, Johannesburg, South Africa, Cas no. 67-64-1), 28 mL of water, and 2 mL of acetic acid (Merk, Johannesburg, South Africa, Cas no. 64-19-7) were mixed (70/28/2). The mixture of the plant material, 70% acidified acetone, and hexane (Merk, Johannesburg, South Africa, Cas no. 110-54-3) were shaken at 200 rpm for 24 h at an ambient temperature. After the 24 h extraction, the hexane fraction was decanted from the 70% acidified acetone fraction. Subsequently, the 70% acetone extract was partitioned into two portions, A and B. Portion A of the 70% acidified acetone fraction was dried and designated as the crude extract, and portion B was consecutively fractionated using liquid–liquid extraction with solvents of increasing polarity, namely, dichloromethane, ethyl acetate, and butanol [59]. The extraction flowchart is represented in Figure 7.

### 3.3. Phytochemical Screening

#### 3.3.1. Determination of Phenolic Content

The Folin–Ciocalteu reagent (Merk, Johannesburg, South Africa, Cas no. F9252) method described by Tambe and Bhambar [60] was adopted to determine the total phenolic contents of different fractions from the leaves and stems. Gallic acid (0.08–1.25 mg/mL) (Merk, Johannesburg, South Africa, Cas no. 149-91-7) was used as a standard for the quantification. The results were expressed as milligrams of gallic acid equivalence/gram of extract (mg GAE/g extract).

#### 3.3.2. Determination of Total Flavonol Content

The aluminium chloride method detailed by Iqbal et al. [61] was followed to determine total flavonol content. Quercetin (Merk, Johannesburg, South Africa, Cas no. 117-39-5) was used as a standard using different concentrations (16–250 µg/mL). Results were expressed as mg quercetin equivalent per gram of extract (mg QE/g).

#### 3.3.3. Determination of Total Proanthocyanidin Content

Total proanthocyanidin content was quantified by following the procedure described by Sun et al. [62]. Gallic acid (Merk, Johannesburg, South Africa, Cas no. 149-91-7) was used as a standard control, and concentrations between 250 and 16 µg/mL were used to construct a standard curve. Total proanthocyanidin content was expressed as milligram gallic acid equivalence/gram of extract (mg GAE/g).

#### 3.3.4. Quantification of Total Terpenoid Content

The total terpenoid content (TTC) of the fractions was determined by the method detailed by Truong et al. [63]. Linalool (Merk, Johannesburg, South Africa, Cas no. 78-70-6) was used to construct a standard curve. The TTC of the extracts was calculated as mg of linalool per gram of extract.

### 3.4. Antioxidant Screening

#### 3.4.1. Free Radical (DPPH) Scavenging Assay

Free radical scavenging activity of the fractions was determined using 2,2-Diphenyl-1-picrylhydrazyl (DPPH) method [64]. Different concentrations of the fractions (15.63–250 µg/mL) were prepared. L-ascorbic acid (Merk, Johannesburg, South Africa, Cas no. 50-81-7) was used as a standard control. To solutions with the fractions, 0.2 mM DPPH (Inqaba biotec, Tshwane, South Africa, Cas no GLS GX8745) was added, and the mixtures were allowed to react in the dark for 30 mins. The solutions were analysed at 517 nm with a UV/VIS spectrophotometer (Thermo Scientific, CAT:840-209800, Waltham, MA, USA, Genesys 10S UV-VIS, Menlo Park, CA, USA).

#### 3.4.2. Hydrogen Peroxide Assay

A solution of hydrogen peroxide (40 mM) (Merk, Johannesburg, South Africa, Cas no. 7722-84-1) was prepared in 0.43 mM phosphate buffer (pH 7.4). Different concentrations (15.63–250 µg/mL) of the fractions (or L-ascorbic acid) were added to a hydrogen peroxide solution (0.6 mL, 40 mM). The absorbance of hydrogen peroxide at 230 nm was determined after 10 min against a blank solution containing phosphate buffer without hydrogen peroxide [65].

### 3.5. Antibacterial Activity

#### 3.5.1. Bacterial Pathogens and Maintenance

*Staphylococcus aureus* (ATCC 29213), *Enterococcus faecalis* (ATCC 29212), *Escherichia coli* (ATCC 28922), *Pseudomonas aeruginosa* (ATCC 27853), and *Mycobacterium smegmatis* (ATCC 1441) were used to assess the antibacterial, anti-motility, and anti-biofilm activities. All the bacterial cultures were grown at 37 °C. The Gram-negative and Gram-positive bacteria were cultured in nutrient broth (Merk, Johannesburg, South Africa, Cas no. 70122). *M. smegmatis* was cultured in Middlebrook 7H9 base (Merk, Johannesburg, South Africa, Cas no. M0178) mixed with glycerol (Inqaba biotec, Tshwane, South Africa, Cas no. 56-81-5) and oleic albumin dextrose catalase (OADC) growth supplement (Merk, Johannesburg, South Africa, Cas no. M0678).

#### 3.5.2. Broth Micro-Dilution Assay

The antibacterial activity of the fractions was evaluated using the broth micro-dilution assay described by Eloff [66]. The stock cultures were grown at 37 °C overnight, and, using standards, working concentrations were adjusted such that *Escherichia coli* (2 × 10^10^ cfu/mL), *Staphylococcus aureus* (2 × 10^8^ cfu/mL), *Mycobacterium smegmatis* (2 × 10^5^ cfu/mL), *Enterococcus faecalis* (3 × 10^8^ cfu/mL), and *P. aeruginosa* (3 × 10^9^ cfu/mL) were used for the bioassay. Sterile distilled water (100 µL) was added to each well of a 96-well microtitre plate. The extracts (10 mg/mL) were serially diluted with distilled water in the 96 well microtitre plates to achieve a concentration of 2.5–0.02 mg/mL in 100 µL of volume. The test bacterial culture (100 μL) was added to respective wells. The microtitre plates were incubated for 24 h at 37 °C. Following incubation, 40 µL of 0.2 mg/mL of p-iodonitrotetrazolium chloride (INT) (Inqaba biotec, Tshwane, South Africa, Cas no. GLS GC3113) was added and incubated for 30 min. Gentamicin (Inqaba biotec, Tshwane, South Africa, Cas no. GLS GA7939) and rifampicin (Inqaba biotec, Tshwane, South Africa, Cas no. GLS GA1853) were used as positive controls for respective bacterial strains. Sterile distilled water was used as the negative control. A visible colour change to pink/red was indicative of viable cells, and the lowest concentration of the fractions that lead to unchanged wells was defined as the MIC.

#### 3.5.3. Growth-Kinetic Curves

The effect of the fractions on the kinetics of bacterial growth was investigated by inoculating overnight cultures of *P. aeruginosa*, *E. coli*, *K. pneumoniae*, *S. aureus* into 20 mL of nutrient broth (Merk, Johannesburg, South Africa, Cas no. 70122), and *M. smegmatis* into OADC supplemented Middlebrook 7H9 broth base. The inoculum was inoculated such that the start OD_600_ at t = 0 is 0.02. Different concentrations (4MIC, 2MIC, MIC, 0.5MIC) of the extracts were added to the flasks. Flasks with the culture only served as a positive control, flasks without both the fractions and culture served as a negative control, and flasks with the fractions and media only served as colour controls for reading absorbance at 660 nm (OD_600_ nm). Readings were taken at 3, 6, 9, 18, 24 h intervals [67].

### 3.6. Anti-Biofilm Screening

#### 3.6.1. Inhibition of Development of Pre-Formed Biofilms

The ability of the fractions to eradicate mature biofilms and prevent further biofilm formation was evaluated. Standardised cultures (OD_600_ = 0.02) of *M. smegmatis*, *S. aureus*, *P. aeruginosa*, *K. pneumoniae*, and *E. coli* were added into 96-well plates and incubated at 37 °C for 48 h at static conditions at final volumes of 100 μL. Following incubation, the biofilms were aspirated with fresh media, and the fractions (100 μL) were added to give final concentrations corresponding with multiples of their respective MICs (4MIC, 2MIC, MIC, 0.5MIC). The plates were further incubated at 37 °C for 24 h. Gentamicin (Inqaba biotec, Tshwane, South Africa, Cas no. GLS GA7939) was used as a positive control, and sterile distilled water was used as negative control [53].

#### 3.6.2. Crystal Violet Staining Assay

To quantify the inhibition of mature biofilms, the treatment plates were washed three times with sterile distilled water and oven-dried at 60 °C for 45 min. The wells were then stained with 100 μL of 0.1% crystal violet (Inqaba biotec, Tshwane, South Africa, Cas no. GA9809) and incubated at room temperature for 15 min. The plates were washed three times with sterile distilled water to remove unabsorbed stains. The crystal violet was solubilised by adding 125 μL of ethanol (Merk, Johannesburg, South Africa, Cas no. 64-17-5), and the solution was transferred to a new plate. The absorbance was measured at 590 nm using a microplate reader (Thermo Scientific, CAT:1530, Multiskan sky, Singapore) [68].

#### 3.6.3. Metabolic Activity of Biofilms

The anti-metabolic activity of the fractions was quantified as described by Mohsenipour and Hassanshahian [69]. The 48 h formed biofilms were washed three times with 1× phosphate-buffered saline. Different concentrations of selected fractions were added microplate was incubated for 24 h at 37 °C. Subsequently, 50 µL of INT (Inqaba biotec, Tshwane, South Africa, Cas no. GLS GC3113) solution was added to each well and incubated in the dark at 37 °C for 30 min. Absorbance was measured at 490 nm with a microplate reader (Thermo Scientific, CAT:1530, Multiskan sky, Singapore). The percentages of reduced biofilm metabolic activity of the treated and untreated biofilms were determined.

### 3.7. Anti-Motility Assay

The swarming motility assay was conducted following the protocol described by Kuchma et al. [70], with slight modifications. A bacteriological agar solution with a concentration of 0.5% was added with nutrient broth, dissolved in distilled water. Bacteriological agar (Merk, Johannesburg, South Africa, Cas no. 9002-18-0) was then mixed with plant extracts at different concentrations (4MIC, MIC, 0.5MIC) and was poured into sterile petri dishes and allowed to solidify. Overnight cultures (10 μL) were inoculated at the centre of the soft-agar plates and incubated at 37 °C for 24 h. Plates without plant extracts were used as a positive control (untreated cultures). Motility was observed as an increase in the diameter of the circle from the point of inoculation after incubation and images were taken.

### 3.8. Cytotoxicity on Artemia Salina

The eggs of brine shrimps were hatched in a laboratory at room temperature. Briefly, 1 g of brine shrimp eggs were added to 1 L of saline water. The saline water was prepared by dissolving 33 g of non-iodised salt in 1 L of distilled water. The pH of the saline water was adjusted to 8.5 using 0.5 M sodium hydroxide (NaOH) (Merk, Johannesburg, South Africa, Cas no. 1310-73-2). The eggs were incubated for 48 h at ambient temperatures. The hatchery apparatus was illuminated during the incubation with a 40 W electric bulb. For the test, plant extracts were serially diluted in freshly prepared saline water to obtain a concentration ranging between 1 µg/mL and 1000 µg/mL up to a volume of 1 mL. The incubation duration was set to 24 h at room temperature. The live brine shrimps were counted visually. The IC_50_ (µg/mL) and selectivity indices were determined [71]. The procedure was carried out in triplicate. The selectivity index values were calculated by dividing cytotoxicity LC_50_ values by the MIC values of the test bacteria in the same units (SI = LC_50_/MIC). Selectivity index values greater than one suggest that extracts are less toxic to the host cell than the bacteria [72]. 

### 3.9. Statistical Analysis

Results were expressed as means ± standard deviation of triplicate determinations. Statistical analysis was performed by IBM Statistical Package for the Social Sciences (SPSS) (version 22, Johannesburg, South Africa) by a two-way analysis of variance (ANOVA) followed by Tukey multiple comparison post hoc test. The significant difference was considered when *p* < 0.05, and conversely, non-significance was indicated when *p* > 0.05.

## 4. Conclusions

*Myrothamnus flabellifolius* plays an important role in ethnomedicine to treat various ailments, including infectious diseases. Here, we report on the antioxidant, cytotoxicity, antibacterial, anti-motility, and anti-biofilm activities of *M. flabellifolius* polyphenols from the leaves and stem parts. The study revealed that the ethyl acetate fractions from both the stem and leaves had comparable antioxidant activity to L-ascorbic acid and broad-spectrum antibacterial activity at non-cytotoxic concentrations. Moreover, these ethyl acetate fractions demonstrated notable anti-motility and anti-biofilm activity that involved eradicating biofilm biomass and inactivating metabolic activity and highlighted the need to broaden our current knowledge of the effects of sub-MICs on biofilm formation. Molecular investigation is required to explore the exact mechanisms of the antibacterial and anti-virulence action and functions of *M. flabellifolius* polyphenols.

## Figures and Tables

**Table 1:** Antioxidant activity of leaf and stem fractions.

Extract	DPPH Radical Scavenging Activity	Hydrogen Peroxide Scavenging Activity
	EC_50_ (µg/mL)	
LCR	35.46 ± 0.90 ^e^	252.65 ± 1.41 ^c^
LDCM	190.40 ± 0.96 ^h^	447.69 ± 3.02 ^g^
LEA	7.57 ± 0.13 ^b^	163.19 ± 0.90 ^a^
LBUT	16.85 ± 1.16 ^c^	232.62 ± 0.58 ^c^
LRW	75.60 ± 0.80 ^f^	761.95 ± 96 ^h^
SCR	33.98 ± 0.96 ^e^	303.4 ± 1.49 ^d^
SDCM	133.70 ± 1.10 ^g^	853.41 ± 1.32 ^i^
SEA	2.99 ± 0.46 ^a^	197.97 ± 0.32 ^b^
SBUT	8.54 ± 0.54 ^b^	291.57 ± 1.15 ^d^
SRW	20.11 ± 0.74 ^d^	247.98 ± 1.11 ^c^
Ascorbic acid	20.41 ± 0.92 ^c,d^	364.2 ± 0.80 ^e^

LCR: leaf crude. LDCM: leaf dichloromethane. LEA: leaf ethyl acetate. LBUT: leaf butanol. LRW: leaf residual water. SCR: stem crude. SDCM: stem dichloromethane. SEA: stem ethyl acetate. SBUT: stem butanol. SRW: stem residual water values expressed as mean ± standard deviation; Tukey multiple comparison post hoc: values with different letter superscripts in a column are significantly different (*p* < 0.05).

**Table 2:** Cytotoxicity, antibacterial activity, and selectivity indices of *M. flabellifolius* leaves and stem fractions against common bacterial pathogens.

Extract	Cytotoxicity (Brine Shrimp Lethality)	*E. coli*		*P. aeruginosa*		*K. pneumoniae*		*S. aureus*		*M. smegmatis*	
	LC_50_ (µg/mL)	MIC (µg/mL)	SI	MIC (µg/mL)	SI	MIC (µg/mL)	SI	MIC (µg/mL)	SI	MIC (µg/mL)	SI
LCR	1107.2 ± 1.30 ^f^	310	3.6	310	3.6	310	3.6	630	1.8	630	1.8
LDCM	979.65 ± 1.4 ^g^	630	1.6	630	1.6	630	1.6	2500	0.4	1250	0.8
LEA	566.57 ± 1.315 ^b^	310	1.8	160	3.5	160	3.5	310	1.8	630	0.9
LBUT	529.79 ± 1.09 ^a^	310	1.7	310	1.7	630	0.8	630	0.4	630	0.8
LRW	822.12 ± 0.87 ^c^	-	ND	1250	0.7	-	ND	2500	0.3	2500	0.3
SCR	1430 ± 1.16 ^h^	630	2.3	310	4.6	630	2.3	1250	1.1	1250	1.1
SDCM	1148.12 ± 1.88 ^g^	1250	0.9	630	1.8	1250	0.9	1250	0.8	2500	0.5
SEA	941.62 ± 0.57 ^d^	310	3.0	160	5.9	310	3.0	310	3.0	630	1.5
SBUT	980.03 ± 1.02 ^e^	310	3.2	630	1.6	630	1.6	630	1.6	630	1.6
SRW	2157.05 ± 0.77 ^i^	-	ND	1250	1.7	-	-	-	ND	-	ND
Gentamicin	5.56 ± 1.16 ^j^	0.2	34.8	0.3	17.9	2.0	2.8	0.9	5.9	ND	ND
Rifampicin	ND	ND		ND		ND		ND		1.6	ND

LCR: leaf crude. LDCM: leaf dichloromethane. LEA: leaf ethyl acetate. LBUT: leaf butanol. LRW: leaf residual water. SCR: stem crude. SDCM: stem dichloromethane. SEA: stem ethyl acetate. SBUT: stem butanol. SRW: stem residual water. Cytotoxicity values expressed as mean ± standard deviation (SD) of triplicates of LC_50_ values; values with different letter superscripts in a column are significantly different (*p* < 0.05); same letter superscript values in a column are not significantly different (*p* > 0.05). (-): no detected activity at concentrations <2.5 mg/mL, ND: not determined.

## Data Availability

Data are contained within the article.
